# Prioritisation of quality indicators for elective perioperative care: a Delphi consensus

**DOI:** 10.1186/s13741-020-0138-7

**Published:** 2020-03-10

**Authors:** D. Gilhooly, M. Chazapis, S. R. Moonesinghe

**Affiliations:** 1 UCL/UCLH NIHR Surgical Outcomes Research Centre, Centre for Perioperative Medicine, Division of Surgery and Interventional Science, Charles Bell House, University College London, London, W1W 7TS UK; 2 Department of Anaesthesia and Perioperative Medicine, University College Hospital, London, NW1 2BU UK; 3 Health Services Research Centre, National Institute for Academic Anaesthesia, Royal College of Anaesthetists, Churchill House, 35 Red Lion Square, London, WC1R 4SG UK

**Keywords:** Structure indicators, Process indicators, Perioperative medicine, Quality measures, Patient care

## Abstract

**Background:**

A systematic review of the peer-reviewed and grey literature previously identified over 1200 perioperative structure and process quality indicators. We undertook a Delphi consensus process with the aim of creating a concise list of indicators that experts deemed most important for assessing quality in perioperative care

**Methods:**

A basic Delphi consensus was completed using an online survey which was distributed to surgeons, anaesthetists, nurses, physicians and lay representatives. Participants were asked to prioritise the indicators in order of importance (high, medium or low) to be included for collection in a national perioperative quality improvement programme.

**Results:**

One hundred and thirty-seven indicators were included in the first iteration of the Delphi consensus (91 structure and 48 process indicators). Sixty-three experts agreed to participate and the consensus was completed in five rounds. Ninety-five indicators were agreed as high priority: 65 structural and 30 process indicators.

**Conclusion:**

The Delphi consensus process was able to reduce the number of recommended indicators to only a modest extent. Further work to evaluate the practicalities of routinely collecting such a comprehensive list of quality indicators is now required.

## Introduction

Evaluation of service quality, in order to identify variation and drive improvement, is becoming increasingly important in healthcare. Quality indicators are measureable elements of practice performance, chosen on the basis of empirical evidence or agreement of experts. Healthcare with its many facets presents its own unique challenges to define and measure quality (Mosadeghrad, 2012). While bodies such as the WHO have developed frameworks (WHO, 2003), perhaps the most commonly used is the model first devised by Avedis Donabedian in 1966 (Donabedian, 1988). This conceptual model breaks down health services into three categories: “structure”, “process” and “outcomes” for assessing quality. Within this framework, indicators of quality may be used. Structural indicators refer to the actual structures there are in place to provide the care such as the building, equipment etc. as well as policies for their care and maintenance. Process indicators refer to the actions that are carried out on the patient during the delivery of care, while outcomes encompass the effects that the healthcare has on the patients.

A systematic review of perioperative process and structure indicators has recently evaluated both research and grey literature databases as well as professional and governmental body publications (Chazapis et al., 2018). This comprehensive review, covering an 11-year period (January 2005–January 2016), identified 1282 clinical indicators, which mainly focused on effectiveness (38%), safety (29%) and efficiency (26%). The majority of the indicators were extracted from clinical practice guidelines, service evaluations and validation studies. Consistent with a previous review of anaesthesia indicators (Haller et al., 2009), this review revealed that the majority of indicators (53%) had no level of evidence ascribed to their literature. Research has already demonstrated that standardization of care in the perioperative period reduces morbidity and mortality in the region of 30 and 50% respectively (Haynes et al., 2009; de Vries et al., 2010). With the identification of these indicators, validation can be achieved through measuring their association with patient-centred outcomes and leading to standards being set for perioperative care.

The aim of this Delphi consensus was to narrow down this list of structural and process indicators identified, to a short-list of quality indicators which are considered most important for assessing perioperative quality. The intention was that these indicators would then be considered for incorporation into the dataset of a national perioperative quality improvement programme (PQIP, www.pqip.org.uk), for major elective non-cardiac surgery to explore compliance in practice and for their validity.

## Methods

A Delphi consensus was performed to develop a hierarchy of structural and process quality indicators to be included in the datasets for PQIP. Research ethics committee approval was not required as this study did not involve patients or their data.

### Formation of 1st iteration of Delphi

The systematic review identified 1282 indicators that were aggregated to 261 as a result of duplication and consisted of 112 structural and 149 process indicators. Indicators were categorized then subdivided into preoperative, intraoperative, postoperative and general (indicators applying to the whole perioperative period) perioperative indicators. Indicators specific to emergency surgery were excluded as not being relevant to an improvement programme based in elective perioperative care. Enhanced Recovery after Surgery (ERAS) indicators were also not considered in the consensus process. Enhanced recovery was implemented in the UK as a result of a national initiative led by the Department of Health. Despite this, compliance with enhanced recovery indicators has been noted to be a variable in previous national audits. (Simpson et al., 2015) Therefore, we deemed the measurement of enhanced recovery indicators to be sufficiently valid that we would not omit these from the PQIP measurement and improvement programme even if the expert consensus was that they were not important.

A steering committee reviewed each indicator and those that could not be easily measured in the context of a large-scale research/improvement programme were removed: for example, ‘appropriate surgical approach’ taken for a procedure (this would most likely require independent case note peer review to be meaningful). Others which were considered to be non-generic were also removed (e.g. that a computed tomography scan should be undertaken and reported before surgery). All references were checked and graded and where multiple existed, the reference with the highest grade was used. Papers were then re-reviewed for the exact wording of each statement to avoid interpretation bias.

The aim of this consensus exercise was to prioritise the quality indicators into high, medium or low priority to be included in a structural questionnaire distributed every 3 years to UK hospitals undertaking elective major surgery, and a list of process indicators to be measured for all patients entered into the PQIP programme. Responses of “Don’t know” and “Outside my area of expertise” were included in the consensus as experts comprised a diverse, heterogeneous group and were allowed to waive a question they felt unable to provide an informed answer, similar to prior studies (Arce et al., 2014).

### Selection of experts

The definition of an expert has been the focus of debate; they can have expertise in the given field or have a vested interest in the results of the study. As a consequence, studies have shown that panel composition can influence ratings (Campbell et al., 1999) and that heterogeneity can lead to better acceptance of outcomes; however, this can lead to difficulty in attaining consensus (Bantel, 1993). Representatives from the Royal College of Anaesthetists, the Association of Anaesthetists of Great Britain and Ireland, the Faculty of Intensive Care Medicine, the Royal College of Surgeons England, the Royal College of Nursing including the Perioperative Association, the Royal College of Physicians, the British Geriatrics Society and lay representatives from the above organisations were invited to participate.

### Definition of consensus

Consensus was defined as 75% or greater agreement for a statement based on a systematic review of quality indicators (Boulkedid et al., 2011). The stability of answers between rounds was based on individual participant stability as opposed to group stability. A statement was considered stable if 80% or greater of the experts did not change their responses between two consecutive responses with a minimum response rate of 80% (Iniyan et al., 1998).

### Piloting

Piloting of the first iteration of the consensus for readability, clarity of meaning and ease of completion revealed a completion time of 45 min. It was decided to have two separate Delphi consensuses, one based on structural and the other on process indicators. The experts would be divided into two balanced groups based on their profession, participating in one Delphi consensus, thus avoiding survey fatigue.

### Distribution of consensus

The representatives from the aforementioned colleges were put forward. An introductory email was sent to each expert describing the background and aim of the consensus along with a link to the consensus hosted on an online platform, Form Assembly (Bloomington, Indiana, USA) (FormAssembly & LLC, 2019). Comment boxes were provided for feedback or to suggest indicators experts felt were missing. Voting was anonymous and each expert’s vote had equal weight in analysis. Experts were given 4 weeks to complete the consensus with reminder emails to encourage completion.

Subsequent iterations of the consensus included the combined results of the group from the previous round under each question. Individual participant results were emailed to the respective experts as a word document attached to the invitation to participate in the next round with the link to the new iteration.

## Results

Of the 112 structural and 149 process indicators, after the removal of indicators specific to ERAS or emergency surgery, and those viewed by the steering group as difficult to assess, 91 structural and 48 process indicators remained. These were used in the first iteration of the Delphi surveys respectively.

Sixty-three participants agreed to participate in the first round and are listed in Table 1.

**Table 1:** Breakdown of participants

Profession	*N* (%)
**Anaesthetist**	19 (30.2%)
**Lay representative**	8 (12.7%)
**Nurse**	2 (3.2%)
**Physician**	12 (19.0%)
**Surgeon**	22 (34.9%)

Feedback from the first iteration of the structural survey revealed that one indicator had been updated since the completion of the systematic review and was therefore changed. Consensus was reached on 113 of the indicators and stability on 21 indicators. Five indicators, 3 structural and 2 processes, had neither reached stability nor neared consensus by the fifth round, the decision to terminate the Delphi was made at this point. The results were then correlated and fed back to participants. Reply rates for each consensus round are shown in Table 2. Final results are in Tables 4 and 5 including individual indicator, priority rating and references (Additional file 1). The majority of indicators were rated as high priority: 65 (71.4%) structure and 30 (62.5%) process indicators.

**Table 2:** Reply rates for each round

	Structural indicators		Process indicators	
	Participants	Replies	Participants	Replies
**Round 1**	32	20 (62.5%)	31	19 (61.3%)
**Round 2**	20	13 (65.0%)	19	15 (78.9%)
**Round 3**	13	12 (92.3%)	15	13 (86.7%)
**Round 4**	12	12 (100%)	13	11 (84.6%)
**Round 5**	12	12 (100%)	11	10 (90.9%)

The aggregate priorities for indicators of each survey are shown in Table 3.

**Table 3:** Results of consensus

	Structural indicators (***n*** = 91)	Process indicators (***n*** = 48)
**High priority**	65	30
**Medium priority**	10	6
**Low priority**	0	0
**Don’t know/Outside my expertise**	2	0
**Stability**	11	10
**No consensus or stability**	3	2

## Discussion

The aim of this Delphi process was to produce a focused list of high priority structural and process indicators which could be measured in a national programme to evaluate the quality of perioperative care. The experts were unable to substantially narrow down the long-list of candidate indicators, rating over 65% of the total as high priority. In the preoperative period, safe, efficient, patient-centred indicators were deemed important to effectively manage comorbidities and patient expectations in order to avoid delays. High priority intraoperative indicators focused on having an adequately resourced operating department and policies in place for maintaining standards of care (e.g. WHO checklist, antibiotic prophylaxis, remote site anaesthesia) and dealing with emergencies. Appropriate levels of care and monitoring of patients in the postoperative period were of high importance for early recognition of complications as well as concise documentation. Looking at the whole perioperative period, consultant numbers, having responsible leads and policies for the main anaesthetic/perioperative sub-specialties and policies for seamless handover of patient care along the perioperative pathway were high priority. Consensus was not achieved on indicators such as numbers of staff per patient number reviewed in pre-assessment clinics, access to interpreters, the design or capacity of PACU or team training. This may be a reflection both of the panel’s views of their importance, and also of the likelihood that these indicators are causally linked to clinical patient outcomes. Other indicators which were deemed unimportant may have been ranked lower due to the lack of empirical evidence supporting their use—one such example is the provision of alternative language leaflets. While not strictly ‘evidence based’, such indicators may still be important for patient experience, and their low ranking may in part be due to the imbalance between clinicians and lay representatives on our Delphi panel. Compliance with some of the quality indicators we evaluated is now so ubiquitous in high income settings (such as transferring patients according to guideline or clippers for hair removal) that there may be little benefit in assessing them for the purpose of stimulating improvement.

Donabedian’s classification for quality measurement describes three categories: structure, process and outcome. The most commonly used outcome indicator is the 30-day mortality, but as mortality in surgery reduces year on year, morbidity may be a better marker of quality, despite the complexities associated with defining and measuring it (Myles et al., 2016). Furthermore, longer-term and more patient-centred outcomes, such as health-related quality of life and disability free survival, are likely to become predominant in both clinical trials and quality measurement systems, but again, these measures have implementation challenges for routine use—particularly cost, the requirement for patient-engagement and the risks associated with various response biases due to issues such as age and socioeconomic status (Dawson et al., 2010; Schamber et al., 2013; Patel et al., 2015). Additionally, the need for the standardising of end points in perioperative trials is fundamental to maximizing the quality of collaborations in this field (Boney et al., 2016). Bearing these issues in mind, structure and process indicators may yet provide useful surrogates for an outcome which can be more easily measured.

Structural indicators are usually easy and inexpensive to assess; however, the evidence base for how these are defined has largely been limited to observational studies rather than trials (Birkmeyer et al., 2004). Making a structural change in healthcare may present a substantial financial, practical and political challenge. The results of our Delphi process for structural indicators placed emphasis on protocols and policies for care of surgical patients. In the preoperative setting, this meant having a responsible physician and policies and pathways to manage patient comorbidities, thereby allowing abnormal results to be flagged in time. Safe care and transfer of patients in the intraoperative period and availability and maintenance of equipment were viewed as important. Cancellation rate measurement is also a good reflection of how good a hospital is at preparing patients and their internal organisation and was thought to be of high priority. Documentation and monitoring in the postoperative period for early detection of morbidity early and the review of outcomes were also high priority. Overall, there was a focus on safe, effective and efficient care of patients. From a patient-centred perspective, having an acute pain service and allowing patients to give feedback on their experience and to have access to clinicians post-discharge were also important. This interestingly puts a focus on more readily actionable indicators which are amenable to assessment and quality improvement processes. Although, on paper, the presence of these structures in a hospital may represent good quality of care, it is important that these policies are enforced and part of normal patient care.

A large number of process measures were identified in the perioperative indicators systematic review, but again, many lacked the high levels of evidence (Chazapis et al., 2018). Process measures are good targets for quality improvement, but it is important for this that they have a direct association with patient-centred outcomes (Boney et al., 2016). When evaluating process indicators, it is key that the patient population and the appropriateness of the measures are defined, thereby preventing overgeneralisation of all surgical procedures and importantly, reflecting the care the patients actually receive (Birkmeyer et al., 2004). There were 48 process measures evaluated in this Delphi consensus. Preoperatively, assessing the timeliness of referral to review, investigation to operation and adequate pre-assessment, including informed risk assessment, were high priority. Intraoperatively safe, effective, patient-centred care was more important to assess than the efficiency of the operating theatre. Postoperatively assessing if the patient is warm, comfortable and oxygenated; that family were informed of the outcome; and that there is a provision of increased care for sicker and more vulnerable patients was high priority. It was clear that safe processes of care were a pervading theme.

From the initial 91 structural and 48 process indicators used in the first iteration of our Delphi process, only 21% of the structural indicators and 52% of the process indicators had any type of empirical evidence supporting their use (Chazapis et al., 2018). Of the 30 process indicators and 65 structural indicators which were viewed as high priority by the Delphi expert group, only 13 (23% of the long-list) and 6 (9% of the long-list) respectively had an empirical evidence supporting them. Over and above being evidence based, there are a number of other attributes of the ideal indicator, and the clinical experts on the panel may have considered these other attributes to be of most importance. These include that the indicator should (Mosadeghrad, 2012) be based on agreed definitions and clearly described, (WHO, 2003) be highly or optimally specific and sensitive, (Donabedian, 1988) demonstrate validity and reliability, (Chazapis et al., 2018) have good discrimination, (Haller et al., 2009) allow comparison and (Haynes et al., 2009) be relevant and actionable (Mainz, 2003). When assessed against these criteria, many indicators can be therefore viewed as imperfect. When indicators are implemented, issues of fidelity, documentation, interpretation and frequency will also arise. A WHO checklist may be done, but is it done in the correct manner? If a patient had an anaesthetic risk assessment done and was informed of the risk, was this accurately documented and did the patients fully understand? An important recognition is that structure and process indicators remain prone to measurement and reporting biases which may be less likely to afflict outcome measures. In the future, the evaluation of the importance of a particular indicator might include not just its empirical evidence base and its face validity, but also the likelihood that it might be subject to measurement error (including gaming)—for example, ticking a box to say that a diabetes risk assessment was done might not be as good a quality indicator as laboratory evidence of a glycated haemoglobin measurement being taken within 3 months of surgery.

### Strengths and limitations

First, there are potential limitations with the use of a Delphi methodology as we deployed it. We used a heterogeneous panel of experts for this consensus to ensure acceptability by stakeholders. While this has the advantage of gathering many different viewpoints, therefore, hopefully giving a more rounded result regarding quality of care (Hong et al., 2010), it can also potentially bias results, as not all stakeholders would have sufficient background knowledge of the full perioperative pathway. For this reason, we included the option of “Don’t know” or “Outside my area of expertise”. Inclusion of patients’ perspectives on quality of care is important but rarely incorporated into clinical indicator development (Haller et al., 2009), and studies can fail to include patient involvement (Cassivi et al., 2008). As patient experience is an important element of the NHS (NHS, 2019), we felt it was necessary to have lay representation in this consensus. Unequal representation of the five main professional groups can be viewed as a limitation, but representation from all five groups was present until the final round. Although the second round of the structures Delphi process was below the median for initial replies in a basic Delphi (87%), our final rounds were in line with prior research and even exceed them for structure indicators with 100% replies in the last two rounds (Boulkedid et al., 2011).

As the systematic review covered a 10-year period, it became evident from the feedback that some indicators had been replaced or updated in clinical practice during this time, but not investigated as markers of quality. Our long-list of indicators was based solely on the results of the previous systematic review and not on expert clinical opinion—therefore, some indicators which may be more clinically relevant may not have been included. Many of the indicators considered may suffer from measurement difficulties—for example, an indicator which states ‘patients and their advocates should understand the risks and outcomes associated with their procedure’—evaluating a patient’s true understanding of the risks and benefits of their surgery would be beyond the scope and feasibility of a large-scale programme.

Finally, the majority of the indicators were deemed to be high priority. This may be as a result not only of the way in which we conducted the prioritisation process, but also because the initial list of indicators from the systematic review had been already edited by us, to include only those which we deemed to be of particular relevance to the PQIP programme in elective major surgery. Our final list of indicators may still be considered too long for routine measurement, and consideration should be given in the future to whether and how a further distillation of this list of measures could be achieved.

## Conclusions

From this Delphi consensus, we have identified 95 structural and process indicators for consideration in the datasets for PQIP. These indicators may additionally be useful measures for local or other national QI or quality assurance programmes.

## Supplementary information

**Additional file 1: Table S1.** Structure Indicator Results. **Table S2.** Process Indicator Results. Description of Data: Individual indicators listed with priority rating and referencing.

## Data Availability

All data generated or analysed during this study are included in this published article (and its supplementary information files).

## Abbreviations

AAGBI: Association of Anaesthetists of Great Britain and Ireland
ACHS: Australian Council on Health Standards
ACSA: Anaesthesia Clinical Services Accreditation
AQI: Anaesthesia Quality Institute
ASA: American Society of Anesthesiologists
ERAS: Enhanced Recovery after Surgery
GPAS: Guidance for the Provision of Anaesthetic Services
HQIP: Healthcare Quality Improvement Partnership
NCEPOD: National Confidential Enquiry into Patient Outcome and Death
NHS: National Health Service
NICE: National Institute for Clinical Excellence
PACU: Post anaesthetic care unit
PQIP: Perioperative Quality Improvement Programme
PQRS: Physician Quality Reporting System
RCoA: Royal College of Anaesthetists
RCoS: Royal College of Surgeons
SCIP: Surgical Care Improvement Project
VTE: Venous thromboembolism
WHO: World Health Organisation
