# Supplementary material for: Prioritisation of quality indicators for elective perioperative care: a Delphi consensus
Source: Perioper Med (Lond). 2020 Mar 10;9:8. doi: 10.1186/s13741-020-0138-7 (PMC7063823; doi:10.1186/s13741-020-0138-7)
Supplement: Supplementary file 1 — Additional file 1: Table S1. Structure Indicator Results. Table S2. Process Indicator Results. Description of Data: Individual indicators listed with priority rating and referencing. [file 13741_2020_138_MOESM1_ESM.docx]

Table 4 Structure Indicators Results

| **Preoperative indicators** | **Priority** | **Source & Evidence** |
| --- | --- | --- |
| 1. There is a consultant anaesthetist with responsibility to lead the anaesthetic pre-operative assessment service, and this is factored into their job plan. Additional consultant anaesthetic input is available as required. | High | RCoA ACSA guidelines 2015  Level N/A |
| 2. There are agreed local policies for preoperative preparation as listed, fasting, investigations, cross-match, thromboprophylaxis, diabetes, latex-allergy, antacid prophylaxis | High | RCoA ACSA guidelines 2015  Level N/A |
| 3. There is a policy in place to ensure that abnormal results of investigations are flagged to the relevant person in a timely manner | High | RCoA ACSA guidelines 2015  Level N/A |
| 4. High risk patients are discussed in regular specialty multidisciplinary team meetings with anaesthetic representation. | High | GPAS 2016  Level N/A |
| 5. There is a policy for patients and/or their advocates to be given information about the possible side effects of pain relief drugs. | Medium | RCoA ACSA guidelines 2015  Level N/A |
| 6. Patients and/or advocates have access to an interpreter | Stability (58.3% High) | RCoA ACSA guidelines 2015  Level N/A |
| 7. Facilities for privacy and confidentiality during pre-operative discussion and examination are provided. | Stability (58.3% Med) | RCoA ACSA guidelines 2015  Level N/A |
| 8. There is an agreed internal policy for referral pathways to other specialties to expedite further investigations. | Stability (66.7% Med) | GPAS 2016  Level N/A |
| Local protocols should determine the grade, experience and competency-based training of the nurse undertaking pre- operative assessments and accompanying the patient to the operating department. For 1,000 patients, the following minimum staffing is required | | |
| 9a. 0.6 registered nurses | Stability (58.3% Outside my expertise) | GPAS 2016  Level N/A |
| 9b. 0.3 healthcare assistants. | Stability (58.3% Outside my expertise) | GPAS 2016  Level N/A |

| **Intraoperative indicator** | **Priority** | **Evidence** |
| --- | --- | --- |
| 1. All patients should have a named and documented supervisory anaesthetic consultant who has overall responsibility for the care of every patient. | High | RCoA ACSA guidelines 2015  Level N/A |
| 2.All equipment used to provide anaesthesia, including monitoring equipment, complies with the recommendations of the AAGBI (Association of Anaesthetists of Great Britain and Ireland). | High | RCoS Standards for unscheduled surgical cases 2011  Level N/A |
| 3. There a planned maintenance and replacement programme for anaesthetic equipment as required. The age equipment of the oldest equipment should be given and written evidence of the replacement programme should be provided. A named consultant oversees the provision of anaesthetic equipment | High | RCoA ACSA guidelines 2015  Level N/A |
| 4. Equipment is available to administer oxygen to all patients undergoing procedures under sedation by anaesthetists. | High | RCoA ACSA guidelines 2015  Level N/A |
| 5. There is specialised equipment for the management of difficult airways in everywhere anaesthesia is given | High | RCoA ACSA guidelines 2015  Level N/A |
| 6. Devices for maintaining or raising the temperature of the patient are available including control of theatre temperature. | High | RCoA ACSA guidelines 2015  Level N/A |
| 7. Defibrillators with cardiac pacing mode are available. | High | RCoA ACSA guidelines 2015  Level N/A |
| 8. Equipment for fluid and blood warming and rapid transfusion are available | High | RCoA ACSA guidelines 2015  Level N/A |
| 9. Blood storage facilities are in close proximity to emergency theatres and contain 0 rhesus negative blood. | High | RCoA ACSA guidelines 2015  Level N/A |
| 10. The hospital has a protocol for major haemorrhage, the protocol is available in all key areas and this should include clinical, laboratory and logistic responses. | High | RCoS Standards for unscheduled surgical care 2011  Level N/A |
| 11. Where sedation is provided by an anaesthetist, there is a policy for the provision of this service including all subspecialty areas and the specifications of the facilities provided. | High | RCoA ACSA guidelines 2015  Level N/A |
| 12. There is a policy for the management of complications of neuraxial blockade. | High | RCoA ACSA guidelines 2015  Level N/A |
| 13. In every site where anaesthesia is given emergency drugs including intralipid, sugammadex and dantrolene are available and in-date supply is maintained. | High | RCoA ACSA guidelines 2015  Level N/A |
| 14 Transfers from theatre to PACU* is done by suitable trained staff under the supervision of an anaesthetist. | High | Vimlati L, EJA 2009  Level 5 |
| 15. Minimum monitoring should be attached to patients before induction of anaesthesia and their use continued until the patient has recovered from the effects of anaesthesia. | High | AAGBI guidelines for perioperative monitoring  2016  Level N/A |
| 16. Presence of properly designed transfer trolleys with oxygen cylinders, masks, tubing, infusion poles, equipment to secure and support airway and assist ventilation, provision of clamps for drainage tubes, protective sides, head down tilt possible | High | Vimlati L, EJA 2009  Level 5 |
| 17. After general or regional anaesthesia, or sedation, all patients recover in a specially designated area which meets AAGBI and DoH guidelines⌘. | High | RCoA ACSA guidelines 2015  Level N/A |
| 18a. Until patients can maintain their airway, breathing and circulation, they are cared for on a one-to-one basis by competent and appropriately trained recovery staff | High | RCoS Standards for unscheduled surgical care 2011  Level N/A |
| 18b. Recovery room staff are appropriately trained in all relevant aspects of postoperative care i.e. trained in recovery, basic pain management and employ guidelines to minimise pain and side effects. | High | RCoA ACSA guidelines 2015  Level N/A |
| 18c. At any given time at least one member of recovery staff present is certified as an advanced life support provider or equivalent and there is an anaesthetist available in addition to their theatre responsibilities. | High | RCoA ACSA guidelines 2015  Level N/A |
| 19. Each PACU* should develop suitable recovery and discharge criteria. | High | Vimlati L EJA 2009  Level 5 |
| 20. The cancellation rate is measured; by cause and preventability. | High | AQI Recommended Indicators 2014  Level N/A |
| 21. The percentage of first cases stating on time in the past 3 months | Medium | AQI Recommended Indicators 2014  Level N/A |
| 22. Drugs intended for regional anaesthesia are stored separately from those for intravenous use. | Stability (50% High) | RCoA ACSA guidelines 2015  Level N/A |
| 23.The turnover time between cases is measured. | Stability (41.7% Med) | Urman R, The Ochsner Journal 2012  Level 3B |
| 24. What access to blood and blood conservation techniques are present (cell salvage or acute normovolaemic dilution). A cell salvage machine and trained staff are available for appropriate patients. | No answer (66.7% Med) | RCoA ACSA guidelines 2015  Level N/A |

| **Postoperative indicators** | **Priority** | **Evidence** |
| --- | --- | --- |
| 1. The patient's condition is continuously monitored in PACU*. | High | ASA Standards for Anaesthesia Care 2014  Level N/A |
| 2. A pain assessment is done with each set of vital signs | High | PQRS Measures 2015  Level N/A |
| 3. There is a system for ordering, storage, recording and auditing of controlled drugs in all post-operative areas where they are used. | High | GPAS 2016  Level N/A |
| 4. The patients have a full and formal handover, with verbal and written instruction. | High | Vimlati L,EJA 2009  Level 5 |
| 5a. There are agreed criteria for discharge from recovery | High | RCoA ACSA guidelines 2015  Level N/A |
| 5b. There is an agreed procedure for the removal of endotracheal tubes and supraglottic airways | High | RCoA ACSA guidelines 2015  Level N/A |
| 6. If critically ill patients are held in recovery due to lack of appropriate facilities elsewhere, this should only occur if level 3 critical care standard of treatment can be provided. | High | GPAS 2016  Level N/A |
| 7. There is a policy for a member of the anaesthetic team to visit patients within 24 hrs following their operation (ASA grade 3,4,5: epidural on ward, invasive monitoring in-situ or as requested by health care worker). | High | RCoA ACSA guidelines 2015  Level N/A |
| 8. Early warning systems are used at ward care | High | AQI Intraoperative 2015  Level N/A |
| 9. There is documentary evidence of morbidity and mortality reviews of all anaesthetic activity and all untoward incidents | High | RCoA ACSA guidelines 2015  Level N/A |
| 10. Patients and supporters are given clear information on discharge from the service and are able to make contact with healthcare professionals for advice and support following discharge | High | RCoS Standards for unscheduled surgical care 2011  Level N/A |
| 11. PACU* equipment includes- pulse oximetry, ECG and NIBP are available at bedside and immediately available are capnograph, 12 lead ECG, nerve stimulator, thermometer. | High | Vimlati L EJA 2009  Level 5 |
| Post anaesthesia documentation should include: | | |
| 12a. Patient evaluation on admission and discharge from post anaesthesia care unit or admission to the intensive care unit | High | ASA Documentation of Anaesthesia Care 2013  Level N/A |
| 12b. A time-based record of vital signs and level of consciousness. | High | ASA Documentation of Anaesthesia Care 2013  Level N/A |
| 12c. A time-based record of drugs administered, their dosage and route of administration | High | ASA Documentation of Anaesthesia Care 2013  Level N/A |
| 12d. Type and counts of intravenous fluids administered, including blood and blood products. | High | ASA Documentation of Anaesthesia Care 2013  Level N/A |
| 12e. Any unusual events including post anaesthesia or post procedural complications. | High | ASA Documentation of Anaesthesia Care 2013  Level N/A |
| 12 f. Post anaesthesia visits. | Medium | ASA Documentation of Anaesthesia Care 2013  Level N/A |
| 13. There is regular training and education of staff (at least every two weeks) | Medium | Vimlati L, EJA 2009  Level 5 |
| 14. Patients have a postoperative MDT consultation and there is presence of a discharge coordinator | Medium | Goossens-Laan C, Urol Int 2011  Level 3B |
| 15. There is a minimum of 30 days follow-up for patients who were on the enhanced recovery pathway for colonic surgery | Stability (41.7% Med) | Wind J, BJoS 2006  Level 1A |
| 16. There is a policy to give patients and carers advice on wound and dressing care | Stability (50% Med/High) | NICE Surgical Site Infection 2013  Level N/A |
| That recovery complies with the following standards: | | |
| 17a. PACU* bed area is 12-15 metres squared per patient. | Outside my expertise | Vimlati L EJA 2009  Level 5 |
| 17b. PACU* capacity- 2 recovery bays for each operating table. | Outside my expertise | Vimlati L EJA 2009  Level 5 |

| **General indicators** | **Priority** | **Evidence** |
| --- | --- | --- |
| 1. There is a consultant lead with the responsibility in the following areas: Resuscitation, Day surgery, acute pain management, obstetrics, emergency anaesthesia, remote sites, ICM, anaesthesia, pre-operative assessment simulator training (if available), airway management | High | RCoA ACSA guidelines 2015  Level N/A |
| 2. There are documented and agreed policies and documentation for the handover of care of the patient from one team to the other throughout the preoperative pathway. | High | RCoA ACSA guidelines 2015  Level N/A |
| 3. There is appropriate facilities for rest are available for on-call/on-duty staff. | High | RCoA ACSA guidelines 2015  Level N/A |
| 4. The number of accredited anaesthesia professionals | High | Weiser T, Lancet 2009 Level 5 |
| 5. The number of accredited surgical professionals | High | Weiser T, Lancet 2009 Level 5 |
| 6. The department has developed a funded and staffed acute pain service or this is in development and agreed as part of the annual plan process | High | RCoA ACSA guidelines 2015  Level N/A |
| 7.The number of theatre there are (excluding radiology suites, dedicated obstetric, minor operations but including day theatre) | High | HQIP Audits 2015  Level N/A |
| 8. That the anaesthetic room and operations theatre conform to Dept. of Health building standards. | High | RCoA ACSA guidelines 2015  Level N/A |
| If policies exist for the following in the hospital: | | |
| 9a. Handover. | High | RCoA ACSA guidelines 2015  Level N/A |
| 9b. Anaesthetic emergencies. | High | RCoA ACSA guidelines 2015  Level N/A |
| 9c. Morbidly obese patients. | High | RCoA ACSA guidelines 2015  Level N/A |
| 9d. Complaints. | High | RCoA ACSA guidelines 2015  Level N/A |
| 9e. Remote site anaesthesia | High | RCoA ACSA guidelines 2015  Level N/A |
| 9 f. End of life care. | High | RCoS Standards for unscheduled surgical care 2011  Level N/A |
| 9 g. Critical care referral | High | RCoA ACSA guidelines 2015  Level N/A |
| 10. There is a resuscitation policy. | High | RCoA ACSA guidelines 2015  Level N/A |
| 11. There is a trained resuscitation team for adults. | High | RCoA ACSA guidelines 2015  Level N/A |
| 12. There is a resuscitation officer responsible for staff training and coordination. | High | RCoA ACSA guidelines 2015  Level N/A |
| 13.A representative range of resuscitation equipment, matching that in use and including mannequins, is available for training purposes. | High | RCoA ACSA guidelines 2015  Level N/A |
| 14. There is a policy for senior clinicians to discuss limits of care as appropriate. This should have clear documentation and be reviewed regularly. | High | RCoA ACSA guidelines 2015  Level N/A |
| 15. The service has mechanisms to receive feedback from patients and supporters. | High | RCoS Standards for unscheduled surgical care 2011  Level N/A |
| 16. There are supervisory consultants freely available to all junior anaesthetists and those they are supervising know their identity, location and how to contact them | High | RCoA ACSA guidelines 2015  Level N/A |
| 17. That the hospital is a tertiary referral centre for any specialist services. | Medium | Courrech Stall EFW, EJSO 2010  Level 2B |
| 18. The hospital size (number of beds) | Medium | Gort M, Social Science & Medicine 2013  Level 4 |
| 19. The average case volume per surgeon | Medium | Courrech Stall EFW, EJSO 2010  Level 1A |
| 20. There is a pharmacist readily available to consult with physicians and nurses over non-critical issues. | Medium | Schifftner T, Journal of the American College of Surgeons 2007  Level 3B |
| 21. The hospital disseminates reports to its community on quality and costs of healthcare services. | Medium | Bilimoria K, JNCI 2009  Level 3B |
| 22. The hospital case volume. | Stability (66.7% High) | Courrech Stall EFW, EJSO 2010  Level 1A |
| 23. Alternative language leaflets are available appropriate to the needs of the local population | Stability (66.7% Med) | RCoA ACSA guidelines 2015  Level N/A |
| 24. There is evidence that there is whole team training for both technical and non-technical skills to deal with emergency situations (Non-Technical- Training that promotes teamwork, with a focus on human factors, effective communication and openness) | No consensus or stability (66.7% Med) | RCoA ACSA guidelines 2015  Level N/A |
| 25. The hospital participates in clinical trials. | No consensus or stability (41.7% Med/Low) | Bilimoria K, JNCI 2009  Level 3B |

Table 5 Process Indicators Results

| **Preoperative indicators** | **Priority** | **Evidence** |
| --- | --- | --- |
| 1. The time from referral to when the patient was first seen by a consultant. | High | HQIP Audit for Cancer Referrals 2015  Level N/A |
| 2. That preassessment was done before the day of surgery. | High | GPAS 2016  Level N/A |
| 3. Patients have an anaesthetic risk assessment performed that informs the process of consent and patient is informed of the risk. | High | RCoA ACSA 2015/NCEPOD Knowing the Risk 2014  Level N/A |
| 4. That there is an up do date preoperative medication list available in the medical records. | High | Bergman S, Surgery 2014, Bergman S, J of Am College of Surgeons  Level 3B |
| 5. If the patient is diabetic that they have pre-op glucose monitoring. | High | Watkins J, Am J or Surgery 2010  Level 2B |
| 6. The time from first diagnostic examination and date of surgery, recommended <6 weeks for cancer surgery. | High | Rosselli EJ of C 2010  Level 4 |
| 7. All patients, on admission, receive an assessment of VTE and bleeding risk using risk assessment criteria. Patients are re-assessed within 24 hours of admission for risk of VTE and bleeding. Patients assessed to be at risk of VTE are offered VTE prophylaxis in accordance with NICE guidance. | High | NICE Quality Standard 3: VTE in adults: reducing the risk in hospital 2010  Level N/A |
| 8. The patient had a pre-operative chlorhexidine shower. | Medium | Keenan JAMA Surg 2014  Level 3B |
| 9. If indicated that a stoma site was marked. | Medium | Information Services Division Scotland 2015  Level N/A |
| 10. The patient was told not to remove hair from the surgical site and to have a pre-operative shower. | Medium | NICE Surgical Site Infection 2013  Level N/A |
| 11. The proportion of cancers patients discussed at an MDT pre-op. | Stability  (72.2% Medium) | Rosselli Del Turco M, EJoC 2010  Level 4 |
| 12. If the patient has a malignancy, whether they will have adjuvant therapy. | Stability (63.3% Low) | Kalish B, J Gastrointest Surg 2013  Level 5 |
| 13. The patient was offered verbal and written information on VTE prevention. | Stability (54% Medium) | NICE Quality Standard 3: VTE in adults: reducing the risk in hospital 2010  Level N/A |
| 14. If indicated that the patient had a pre-op assessment by stoma care nurse. | No consensus or stability (72.7% Medium) | Information Services Division Scotland 2015  Level N/A |
| 15. There is performance of risk assessment for pressure ulcers using a standardized scale upon admission. | No consensus or stability (63.6% High) | Bergman S, Surgery 2014; Bergman S, J of Am College of Surgeons  Level 3B |

| **Intraoperative indicators** | **Priority** | **Evidence** |
| --- | --- | --- |
| 1. The WHO checklist is done. | High | Bergman S Surgery 2014, Bergman S J of Am College of Surgeons  Level 3B |
| 2. Surgical procedures with a predicted mortality >10% should be conducted under direct supervision of consultant surgeon and anaesthetist (unless responsible consultants have satisfied themselves that their delegated staff have adequate competency and experience). | High | RCoS The Higher Risk General Surgical Patient 2011  Level N/A |
| 3. An appropriate antibiotic is given as per local guidelines. | High | SCIP Inf-2 guideline  Information Services Division Scotland 2015  Level N/A |
| 4. The measurement and documentation of pain intensity scores after major surgery. | High | ACHS Aus. Clinical Indicator Report 2013  Information Services Division Scotland 2015  Level N/A |
| 5. Intraoperative blood loss is measured and recorded. | High | Bilimoria K, JNCI 2009  Level 3B |
| 6a. If the patient received an intraoperative blood transfusion. | High | Ball C, HPB 2010  Level 3B |
| 6b. If the patient received an unanticipated transfusion greater than 10 units of any blood products. | High | AQI Recommended indicators 2014  Information Services Division Scotland 2015  Level N/A |
| 7. Surgical pathology specimens are correctly labelled: Labelled, filled containers, correct laterality, correct tissue type, patient name, and correct patient name. | High | Makary M, Surgery 2007  Level 2B |
| 8. If the operating time was longer than anticipated. | Medium | Gastmeier P, Infection 2011  Level 3B |
| 9. The operating time is recorded. | Stability (53.8% High) | Gastmeier P, Infection 2011  Level 3B |
| 10. The turnover time between cases. | Stability (54.5% Medium) | Urman R, The Oschner J 2012  Level 3B |
| 11. Surgery patients have appropriate hair removal: use clippers and not a razor. | Stability (54.5% High) | SCIP-Inf 6 guideline  Level N/A |

| **Postoperative indicators** | **Priority** | **Evidence** |
| --- | --- | --- |
| 1. The patient was normothermic post-operatively 36-38 degrees Celsius. | High | Wick E, Dis. Of the colon and Rectum 2008  Level 1B |
| 2. Whether all patients are given supplemental oxygen post-operatively. | High | Vimlati L, EJA 2009  Level 5 |
| 3. Immediately post-surgery a member of the medical/nursing team updates the patient’s supporter(s) of the outcome of surgery. | High | RCoS Standards for unscheduled surgical care 2011  Level N/A |
| 4. An official PACU to ward handover for the patient was performed. | High | Vimlati L, EJA 2009  Level 5 |
| 5. Patients over 65 yrs old are screened for post-operative delirium for the first 3 post-op days. | High | Bergman S, 2013 Journal American College of Surgeons  Level 3B |
| 6a. Patients with mortality risk >10% should be admitted to critical care. | High | RCoS Higher Risk General Surgical Patient 2011  Level N/A |
| 6b. If critical care admission is not possible and that the decision to operate is being made without the provision of appropriate care package: that this is communicated to the patient. | High | NCEPOD Knowing the Risk 2014  Level N/A |
| 7. The discharge destination is recorded. | High | Lemmens L, J of Evaluation in Clinical Practice 2008  Level 3A |
| 8a. Arrangements are in place for the multidisciplinary management of vulnerable elderly patients. | High | RCoA ACSA guidelines 2015  Level N/A |
| 8b. There is routine daily input from Medicine for the Care of Older, people should be available to elderly patients undergoing surgery and is integral to inpatient care pathways in this population. | High | NCEPOD Elderly people undergoing surgery 2010  Level N/A |
| 9. Pain should be brought under control in 48 hrs post-operatively. | High | PQRS Measures 2015  Level N/A |
| That it is recorded if the patient had: | | |
| 10a. A discharge needs assessment. | High | Bergman S, J of Am Col of Surgeons 2013  Level 3B |
| 10b. VTE prophylaxis. | High | Haller G  Anesthesiology  Level 1A |
| 10c. A follow-up phone call in 7 days of discharge. | Medium | ACHS Aus Clinical Indicator Report 2013  Level N/A |
| 11. Daily visible phlebitis scores were done. | Stability (72.7% Low) | Kreckler S, Annals of Surgery 2009  Level 2B |
| 12. If patients are receiving prescribed antiemetic treatment when nausea and vomiting are present during acute pain management. | Stability (72.2% Medium) | Haller G, Anaesthesiology 2009  Level 4 |
| 13. The PACU length of stay is recorded and should not be longer than 24 hrs. | Stability (54% Medium) | Vimlati L, EJA 2009  Level 5 |

| **General indicators** | **Priority** |  |
| --- | --- | --- |
| 1. The national policy for patient identification is followed. Evidence that patients are labelled, that labels are replaced and that patient name and number are both used at every stage of the WHO process (all checks) should be seen | High | RCoA ACSA guidelines 2015  Level N/A |
| 2. Anaesthetists offering perioperative analgesia services should provide, in collaboration with others as appropriate, patient and family education regarding their important roles in achieving comfort, reporting pain, and in proper use of the recommended analgesic methods. | High | ASA Acute pain management in the perioperative setting 2012  Level N/A |
| 3. If the patients had fast track care. | Medium | Dikken J, Ann Surg Oncol 2013  Level 1A |
| 4. The patient is transferred according to AAGBI guidelines. | Stability (63% Medium) | RCoS Standards for unscheduled surgical care 2011  Level N/A |
